# A computational study of exact subgraph based SDP bounds for Max-Cut, stable set and coloring

**DOI:** 10.1007/s10107-020-01512-2

**Published:** 2020-05-25

**Authors:** Elisabeth Gaar, Franz Rendl

**Affiliations:** Institut für Mathematik, Alpen-Adria-Universität Klagenfurt, Universitätsstr. 65-67, 9020 Klagenfurt, Austria

**Keywords:** Semidefinite programming, Relaxation hierarchy, Max-Cut, Stable set, Coloring, 90C22, 90C27

## Abstract

The “exact subgraph” approach was recently introduced as a hierarchical scheme to get increasingly tight semidefinite programming relaxations of several NP-hard graph optimization problems. Solving these relaxations is a computational challenge because of the potentially large number of violated subgraph constraints. We introduce a computational framework for these relaxations designed to cope with these difficulties. We suggest a partial Lagrangian dual, and exploit the fact that its evaluation decomposes into several independent subproblems. This opens the way to use the bundle method from non-smooth optimization to minimize the dual function. Finally computational experiments on the Max-Cut, stable set and coloring problem show the excellent quality of the bounds obtained with this approach.

## Introduction

The study of NP-hard problems has led to the introduction of various hierarchies of relaxations, which typically involve several levels. Moving from one level to the next the relaxations get increasingly tighter and ultimately the exact optimum may be reached, but the computational effort grows accordingly.

Among the most prominent hierarchies are the polyhedral ones from Boros, Crama and Hammer [5] as well as the ones from Sherali and Adams [32], Lovász and Schrijver [25] and Lasserre [22] which are based on semidefinite programming (SDP). Even though on the starting level they have a simple SDP relaxation, already the first nontrivial level in the hierarchy requires the solution of SDPs in matrices of order $$\left( {\begin{array}{c}n\\ 2\end{array}}\right) $$ and on level *k* the matrix order is $n^{O(k)}$. Hence they are considered mainly as theoretical tools and from a practical point of view these hierarchies are of limited use.

Not all hierarchies are of this type. In [5] a polyhedral hierarchy for the Max-Cut problem is introduced which maintains $$\left( {\begin{array}{c}n\\ 2\end{array}}\right) $$ variables at all levels, with a growing number of constraints. More recently, Adams, Anjos, Rendl and Wiegele [1] introduced a hierarchy of SDP relaxations which act in the space of symmetric $n \times n$ matrices and at level *k* of the hierarchy all submatrices of order *k* have to be “exact” in a well-defined sense, i.e. they have to fulfill an *exact subgraph constraint* (ESC).

It is the main purpose of this paper to describe an efficient way to optimize over level *k* of this hierarchy for small values of *k*, e.g. $k\leqslant 7$, and demonstrate the efficiency of our approach for the Max-Cut, stable set and coloring problem. These investigations were started in [12, 13] and here we offer the full picture.

Maintaining $$\left( {\begin{array}{c}n\\ k\end{array}}\right) $$ possible ESCs in an SDP in matrices of order *n* is computationally infeasible even for $k=2$ or $k=3$, because each ESC creates roughly $$\left( {\begin{array}{c}k\\ 2\end{array}}\right) $$ additional equality constraints and at most $2^k$ additional variables.

We suggest the following ideas to overcome this difficulty. First we proceed iteratively, and in each iteration we include only (a few hundred of) the most violated ESCs. More importantly, we propose to solve the dual of the resulting SDP. The structure of this SDP with ESCs admits a reformulation of the dual in the form of a non-smooth convex minimization problem with attractive features. First, any dual solution yields a valid bound for our relaxations, so it is not necessary to carry out the minimization to optimality. Secondly, the dual function evaluation decomposes into two independent problems. The first one is simply a sum of max-terms (one for each ESC), and the second one consists in solving a “basic” SDP, independent of the ESCs. The optimizer for this second problem also yields a subgradient of the objective function. With this information at hand we suggest to use the bundle method from non-smooth convex optimization. It provides an effective machinery to get close to a minimizer in few iterations.

As a result we are able to get near optimal solutions where all ESCs for small values of *k* ($k \leqslant 7$) are satisfied up to a small error tolerance. Our computational results demonstrate the practical potential of this approach.

The paper is organized as follows. In Sect. 2 we briefly describe the Max-Cut, the stable set and the coloring problem along with their semidefinite relaxations, which are well-studied in the literature. Sect. 3 recalls the exact subgraph hierarchy, described in [1]. We introduce a unified setting for all these problems and take a look at their structural properties. In Sect. 4 we reformulate the SDP and consider a partial Lagrangian dual. It results in many subproblems, separating the basic SDP part from the ESC part. The bundle method from non-smooth optimization is described in Sect. 5 as an attractive algorithmic framework to deal with the subproblems in the partial Lagrangian dual. In Sect. 6 we describe our algorithm in order to obtain exact subgraph based SDP bounds. We argue in Sect. 7 that standard SDP solvers are only of limited use when dealing with our ESC hierarchy and present extensive computational results. Finally we close with conclusions and future work in Sect. 8.

We finish this introductory section with some notation. We denote the vector of all-ones of size *n* with $\mathbb {1}_{n}$ and ${\varDelta }_n = \{x \in {{\mathbb {R}}}^{n}_{+}: \sum _{i=1}^{n}x_{i} = 1\}$. If the dimension is clear from the context we may omit the index and write $\mathbb {1}_{}$ and ${\varDelta }$. Furthermore let $N = \{1, 2, \dots , n\}$. A graph *G* on *n* vertices has vertex set *N* and edge set *E*. The complement graph ${\overline{G}}$ of a graph *G* has the same vertex set *N* and contains an edge $\{i,j\} \subseteq N$ if and only if $\{i,j\} \not \in E$. ${{\mathcal {S}}}_{n}$ is the set of *n*-dimensional symmetric matrices. A spectrahedron is a set that is obtained as the intersection of the cone of positive semidefinite matrices with some linear affine subspace.

## Combinatorial problems and semidefinite relaxations

### The Max-Cut problem

In the Max-Cut problem a symmetric matrix $L \in {{\mathcal {S}}}_{n}$ is given and $c \in \{-1,1 \}^{n}$ which maximizes $c^{T}Lc$ should be determined.

If the matrix *L* corresponds to the Laplacian matrix of a (edge-weighted undirected) graph *G*, this is equivalent to finding a partition of the vertices of *G* into two subsets such that the total weight of the edges joining these two subsets is maximized. Such an edge set is also called a *cut* in *G*.

Partitions of *N* into two subsets can be expressed as $c \in \{-1,1 \}^{n}$ where the two subsets of *N* correspond to the entries of *c* with the same sign. Given $c \in \{-1,1\}^{n}$ we call $C=cc^{ {T} }$ a *cut matrix*. The convex hull of all cut matrices (of order *n*) is denoted by

$$\begin{aligned} {{\,\mathrm{CUT}\,}}_{n} = {{\,\mathrm{conv}\,}}\left\{ cc^{ {T} }:~ c \in \{-1,1\}^{n} \right\} \end{aligned}$$

or simply ${{\,\mathrm{CUT}\,}}$ if the dimension is clear from the context. Since $c^{ {T} }Lc = \langle L, cc^{ {T} } \rangle$ the Max-Cut problem can also be written as the following (intractable) linear program

$$\begin{aligned} z_{mc} = \max \{ \langle L, X\rangle :~ X \in {{\,\mathrm{CUT}\,}}\}. \end{aligned}$$

${{\,\mathrm{CUT}\,}}$ is contained in the spectrahedron

$$\begin{aligned} {{\mathcal {X}}}^{E}= \left\{ X \in {{\mathcal {S}}}_{n} : {{\,\mathrm{diag}\,}}(X) = \mathbb {1}_{n},\, X \succcurlyeq 0 \right\} , \end{aligned}$$

hence

1 $$\begin{aligned} r_{mc} = \max \left\{ \langle L,X\rangle :~ X \in {{\mathcal {X}}}^{E}\right\} \end{aligned}$$

is a basic semidefinite relaxation for Max-Cut. This model is well-known, attributed to Schrijver and was introduced in a dual form by Delorme and Poljak [8]. It can be solved in polynomial time to a fixed prescribed precision and solving this relaxation for $n=1000$ takes only a few seconds.

It is well-known that the Max-Cut problem is NP-hard. On the positive side, Goemans and Williamson [14] show that one can find a cut in a graph with nonnegative edge weights of value at least 0.878$z_{mc}$ in polynomial time.

### The stable set problem

In the stable set problem the input is an unweighted graph *G*. We call a subset of the vertices *stable*, if no two vertices are adjacent. Moreover we call a vector $s \in \{0,1 \}^n$ a *stable set vector* if it is the incidence vector of a stable set. The convex hull of all stable set vectors of *G* is denoted with ${{\,\mathrm{STAB}\,}}(G)$. In the stable set problem we want to determine the *stability number* $\alpha (G)$, which denotes the cardinality of a largest stable set in *G*, hence

$$\begin{aligned} \alpha (G) = \max \left\{ \mathbb {1}_{}^{ {T} }s:~ s \in {{\,\mathrm{STAB}\,}}(G) \right\} . \end{aligned}$$

Furthermore we denote with

$$\begin{aligned} {{\,\mathrm{STAB}\,}}^{2}(G) = {{\,\mathrm{conv}\,}}\left\{ ss^{ {T} }:~ s \in {{\,\mathrm{STAB}\,}}(G) \right\} \end{aligned}$$

the convex hull of all *stable set matrices* $ss^{ {T} }$. Then with the arguments of Gaar [12] it is easy to check that

$$\begin{aligned} \alpha (G) = \max \left\{ {{\,\mathrm{trace}\,}}(X):~ X \in {{\,\mathrm{STAB}\,}}^2(G) \right\} . \end{aligned}$$

Furthermore ${{\,\mathrm{STAB}\,}}^{2}(G)$ is contained in the following spectrahedron

$$\begin{aligned} {{\mathcal {X}}}^{S}= \left\{ X \in {{\mathcal {S}}}_{n} :~ X_{ij}=0 \quad \forall \{i,j\} \in E,~ x = {{\,\mathrm{diag}\,}}(X),~ \left( \begin{array}{cc} 1 &{} x^{ {T} } \\ x &{} X \end{array} \right) \succcurlyeq 0 \right\} , \end{aligned}$$

which is known as the *theta body* in the literature. Therefore

2 $$\begin{aligned} \vartheta (G)= \max \left\{ {{\,\mathrm{trace}\,}}(X):~ X \in {{\mathcal {X}}}^{S}\right\} \end{aligned}$$

is a relaxation of the stable set problem. The Lovász theta function $\vartheta (G)$ was introduced in a seminal paper by Lovász [24]. We refer to Grötschel, Lovász and Schrijver [15] for a comprehensive analysis of $\vartheta (G)$.

Determining $\alpha (G)$ is again NP-hard. Contrary to Max-Cut, which has a polynomial time .878-approximation, for every $\varepsilon >0$ there can be no polynomial time algorithm that approximates $\alpha (G)$ within a factor better than $O(n^{1-\varepsilon })$ unless $P=NP$, see Håstad [17].

### The vertex coloring problem

The coloring problem for a given graph *G* consists in determining the *chromatic number* $\chi (G)$, which is the smallest *t* such that *N* can be partitioned into *t* stable sets. Let $S=(s_{1}, \ldots , s_{k})$ be a matrix where each column $s_i$ is a stable set vector and the corresponding stable sets partition *N* into *k* sets. Let us call such matrices *S* *stable-set partition matrices* (SSPM) and denote by |*S*| the number of columns of *S* or equivalently the number of stable set vectors of *S*. The $n \times n$ matrix $X=SS^{T}$ is called *coloring matrix*. The convex hull of the set of all coloring matrices of *G* is denoted by

$$\begin{aligned} {{\,\mathrm{COL}\,}}(G) = {{\,\mathrm{conv}\,}}\left\{ X:~ X \text { is a coloring matrix of }G \right\} . \end{aligned}$$

We also need the *extended coloring polytope*

$$\begin{aligned} {{\,\mathrm{COL}\,}}^{\varepsilon }(G) = {{\,\mathrm{conv}\,}}\left\{ \left( \begin{array}{cc} k &{} \mathbb {1}_{}^{ {T} }\\ \mathbb {1}_{} &{} X \end{array}\right) = \sum _{i=1}^{k} \left( {\begin{array}{c}1\\ s_{i}\end{array}}\right) \left( {\begin{array}{c}1\\ s_{i}\end{array}}\right) ^{ {T} } : \begin{array}{c} S = (s_{1}, \ldots , s_{k}) \text { is a} \\ \text {SSPM of } G,~ X = SS^{ {T} } \end{array} \right\} . \end{aligned}$$

The difficult set ${{\,\mathrm{COL}\,}}^{\varepsilon }$ can be relaxed to the easier spectrahedron

$$\begin{aligned} {{\mathcal {X}}}^{C}= \left\{ \left( \begin{array}{cc} t &{} \mathbb {1}_{}^{ {T} }\\ \mathbb {1}_{} &{} X \end{array}\right) \succcurlyeq 0:~ {{\,\mathrm{diag}\,}}(X)=\mathbb {1}_{n}, X_{ij}=0 ~\forall \{i,j\} \in E \right\} \end{aligned}$$

and we can consider the semidefinite program

3 $$\begin{aligned} t^{*}(G) = \min \left\{ t:~ \left( \begin{array}{cc} t &{} \mathbb {1}_{}^{ {T} }\\ \mathbb {1}_{} &{} X \end{array}\right) \in {{\mathcal {X}}}^{C}\right\} . \end{aligned}$$

Obviously $t^{*}(G) \leqslant \chi (G)$ holds because the SSPM *S* consisting of $\chi (G)$ stable sets yields a feasible coloring matrix $X=SS^{ {T} }$ with objective function value $\chi (G)$. It is in fact a consequence of conic duality that $t^{*}(G)= \vartheta ({\overline{G}})$ holds.

It is NP-hard to find $\chi (G)$, to find a 4-coloring of a 3-colorable graph [16] and to color a *k*-colorable graph with $O(k^{\frac{\log k}{25}})$ colors for sufficiently large *k*, [20].

## Exact subgraph hierarchy

### Definition of the hierarchy

In this section we discuss how to systematically tighten the relaxations (1), (2) and (3) with “exactness conditions” imposed on small subgraphs. We obtained the relaxations by relaxing the feasible regions ${{\,\mathrm{CUT}\,}}$, ${{\,\mathrm{STAB}\,}}^{2}$ and ${{\,\mathrm{COL}\,}}$ of the integer problem to simple spectrahedral sets. Now we will use small subgraphs to get closer to the feasible regions of the original problems again.

For $I \subseteq N$ let $k_{I}=|I|$ be the cardinality of *I*. Furthermore let $G_{I}$ be the induced subgraph of *G* on the set of vertices *I*. If *X* is the $n \times n$ matrix from the relaxations (1), (2) or (3), then we denote with $X_{I}$ the principal $k_{I}\times k_{I}$ submatrix of *X* corresponding to the rows and columns in *I*. Note that $X_{I}$ is the submatrix of *X* corresponding to $G_{I}$.

We first look at the exact subgraph relaxations for Max-Cut. Adams, Anjos, Rendl and Wiegele [1] introduced additional constraints for the Max-Cut relaxation (1) in the following way. The *exact subgraph constraint* (ESC) for $I \subseteq N$ requires that the matrix $X_{I}$ corresponding to the subgraph $G_{I}$ lies in the convex hull of the cut matrices of $G_{I}$, that is

$$\begin{aligned} X_{I}\in {{\,\mathrm{CUT}\,}}_{|I|}. \end{aligned}$$

The ESC for *I* can equivalently be phrased as

$$\begin{aligned} X_{I}= \sum _{i=1}^{t_{I}} \lambda _{i}C^{I}_{i} \end{aligned}$$

for some $\lambda \in {\varDelta }_{t_{I}}$ where $C^{I}_{i}$ is the *i*-th cut matrix of the subgraph $G_{I}$ and $t_{I}$ is the total number of cut matrices. If *X* is a solution of (1) that fulfills the ESC for some *I* we say that *X* is *exact* on *I* and *X* is *exact* on $G_{I}$.

Now we want the ESCs to be fulfilled not only for one but for a certain selection of subgraphs. We denote with *J* the set of subsets *I*, on which we require *X* to be exact, and get the following SDP relaxation with ESCs for Max-Cut.

4 $$\begin{aligned} \max \{\langle L,X\rangle :~ X \in {{\mathcal {X}}}^{E},~ X_{I}\in {{\,\mathrm{CUT}\,}}_{|I|} ~ \forall I \in J \} \end{aligned}$$

Before we give theoretical justification that (4) is worth to be investigated, we present the ESCs for the other problems. We start with the stable set problem on a graph *G* and its relaxation (2). In this case the ESC for $I \subseteq N$, and hence for the subgraph $G_{I}$, requires that $X_{I}\in {{\,\mathrm{STAB}\,}}^{2}(G_{I})$ holds and the SDP with ESCs for the stable set problem is

5 $$\begin{aligned} \max \{ {{\,\mathrm{trace}\,}}(X):~ X \in {{\mathcal {X}}}^{S},~ X_{I}\in {{\,\mathrm{STAB}\,}}^{2}(G_{I}) ~ \forall I \in J \}. \end{aligned}$$

Turning to the coloring problem, we analogously impose additional ESCs of the form $X_{I}\in {{\,\mathrm{COL}\,}}(G_I)$ to obtain the SDP with ESCs

6 $$\begin{aligned} \min \left\{ t:~ \left( \begin{array}{cc} t &{} \mathbb {1}_{}^{ {T} }\\ \mathbb {1}_{} &{} X \end{array}\right) \in {{\mathcal {X}}}^{C},~ X_{I}\in {{\,\mathrm{COL}\,}}(G_{I}) ~ \forall I \in J \right\} . \end{aligned}$$

We now want to investigate the properties of (4), (5) and (6). Towards that end we define the *k*-th level of the *exact subgraph hierarchy* according to [1] by using $J = \{I \subseteq N:~ |I| = k\}$ in the SDPs (4), (5) and (6), respectively. We denote the corresponding objective function values with $z_{mc}^{k}$, $z_{ss}^{k}$ and $z_{c}^{k}$. So in other words the *k*-th level of the exact subgraph hierarchy is obtained by forcing all subgraphs on *k* vertices to be exact in the basic SDP relaxation.

Note that

$$\begin{aligned}&z_{mc} = z_{mc}^{n} \leqslant \dots \leqslant z_{mc}^{k} \leqslant z_{mc}^{k-1} \leqslant \dots \leqslant z_{mc}^{2} \leqslant z_{mc}^{1} = r_{mc}\\&\alpha (G) = z_{ss}^{n} \leqslant \dots \leqslant z_{ss}^{k} \leqslant z_{ss}^{k-1} \leqslant \dots \leqslant z_{ss}^{2} \leqslant z_{ss}^{1} = \vartheta (G) \end{aligned}$$

holds for all $k \in \{2, \dots , n\}$, see [1, 12]. Hence (4) and (5) are relaxations of Max-Cut and the stable set problem.

Furthermore it can be verified that

$$\begin{aligned} t^{*}(G) = z_{c}^{1} \leqslant z_{c}^{2} \leqslant \dots \leqslant z_{c}^{k-1} \leqslant z_{c}^{k} \leqslant \dots \leqslant z_{c}^{n} \leqslant \chi (G) \end{aligned}$$

holds for all $k \in \{2, \dots , n\}$, so for the coloring problem we do not necessarily reach $\chi (G)$ at the *n*-th level. However, the following holds. Let $z_{c\varepsilon }^{k}$ be the optimal objective function value if we add the inequalities $t \geqslant \sum _{i=1}^{t_{I}}[\lambda _{I}]_{i}|S^{I}_{i}|$ where $\lambda _{I}\in {\varDelta }_{t_{I}}$ is a variable for the convex combination for each subgraph $G_{I}$ to the SDP for $z_{c}^{k}$. Then $z_{c\varepsilon }^{n} = \chi (G)$ holds. Hence $z_{c}^{k}$ is a relaxation of $z_{c\varepsilon }^{k}$, which is in turn a relaxation of the coloring problem. As a result it is clear that it makes sense to investigate (4), (5) and (6).

Note that in the case of the stable set and the coloring problem the polytopes ${{\,\mathrm{STAB}\,}}^{2}(G_{I})$ and ${{\,\mathrm{COL}\,}}(G_{I})$ depend on the subgraph $G_{I}$, whereas in Max-Cut the polytope ${{\,\mathrm{CUT}\,}}_{|I|}$ only depends on the number of vertices of $G_{I}$.

Finally let us mention that an important feature of this hierarchy is that the size of the matrix variable remains *n* or $n+1$ on all levels of the hierarchy. On higher levels the ESCs are included into the SDPs in the most natural way through convex combinations. Hence on higher levels of the exact subgraph hierarchy new variables and linear constraints representing convex hull conditions are added to the SDP of the basic SDP relaxation.

Therefore it is possible to approximate $z_{mc}^{k}$, $z_{ss}^{k}$ and $z_{c}^{k}$ by forcing only some subgraphs of order *k* to be exact. This is our key ingredient to computationally obtain tight bounds on $z_{mc}$, $\alpha (G)$ and $\chi (G)$ and also a major advantage over several other SDP based hierarchies [22, 25, 32] for NP-hard problems.

### Structural differences of the three problems

The focus of this paper lies in computational results, so we omit further extensive theoretical investigations, but we want to draw the attention to a major structural difference between the Max-Cut problem and the stable set and the coloring problem. Towards this end we consider one graph from the Erdős-Rényi model *G*(*n*, *p*) with $n=100$ and $p = 0.15$. A graph from this model is a random graph of order *n*, in which each edge appears with probability *p*.

We compute the optimal solutions of the basic relaxations (1), (2) and (3) and denote them by $X^*$. Then for each subgraph $G_{I}$ of order $k\in \{2,3,4,5\}$ we compute the projection distance $\delta _{mc}^I$, $\delta _{ss}^I$ and $\delta _{c}^I$ of the submatrix $X_{I}^*$ of the corresponding $X^*$ to ${{\,\mathrm{CUT}\,}}_k$, ${{\,\mathrm{STAB}\,}}(G_{I})$ and ${{\,\mathrm{COL}\,}}(G_{I})$, respectively. So for example

$$\begin{aligned} \delta _{mc}^I = \min _{C \in {{\,\mathrm{CUT}\,}}_k} \left\Vert X_{I}^*- C \right\Vert , \end{aligned}$$

where $\left\Vert . \right\Vert$ denotes the Frobenius norm. We consider a subgraph $G_{I}$ as violated, if the projection distance is larger than the small tolerance $5\cdot 10^{-5}$.

**Table 1:** The percentage of violated subgraphs of order *k* for one random graph

*k*	2	3	4	5
# Subgraphs	4950	161700	3921225	75287520
% Violated subgraphs MC	0.00	49.59	91.69	99.83
% Violated subgraphs SS	7.54	21.96	41.00	60.88
% Violated subgraphs CO	5.82	16.83	31.90	49.14

**Fig. 1:**
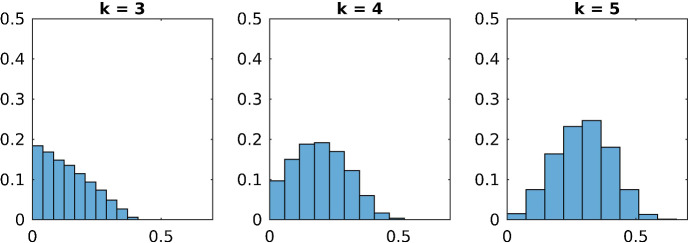
Histogram of $\delta _{mc}^I$ for all violated subgraphs $G_{I}$ of order $k\in \{3,4,5\}$

**Fig. 2:**
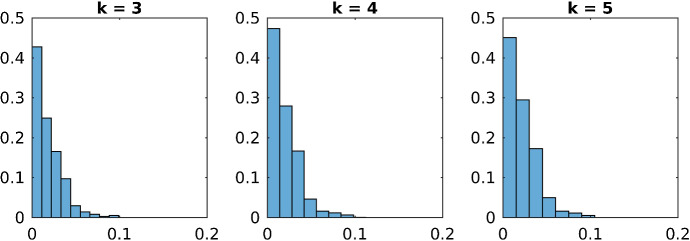
Histogram of $\delta _{ss}^I$ for all violated subgraphs $G_{I}$ of order $k\in \{3,4,5\}$

**Fig. 3:**
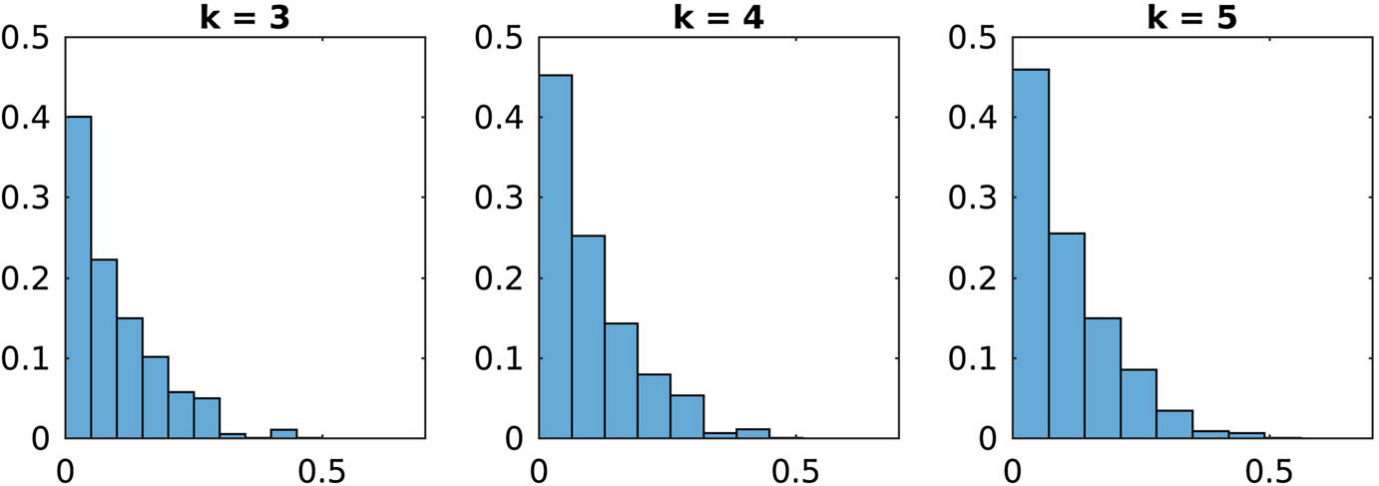
Histogram of $\delta _{c}^I$ for all violated subgraphs $G_{I}$ of order $k\in \{3,4,5\}$

In Table 1 one sees that the number of violated subgraphs is much higher in the case of the Max-Cut problem than for the stable set and the coloring problem. Figures 1, 2 and 3 show the distribution of the projection distances of the violated subgraphs. They are normalized in such a way that 1 is the total number of violated subgraphs. Here it becomes obvious that for the Max-Cut problem most of the violated subgraphs have a large violation, whereas most of the violated subgraphs for the coloring problem have a small violation and an even smaller violation for the stable set problem.

Therefore in the case of the Max-Cut problem there are very many violated subgraphs, and typically all of them have a large projection distance. On the other hand for the stable set and the coloring problem only very few subgraphs have a large projection distance, the majority of the subgraphs is either not violated at all or only violated a little bit. Hence finding significantly violated subgraphs is much more difficult for the stable set and the coloring problem, than it is for the Max-Cut problem.

A possible explanation for this consists of the following dimension argument. Let *G* be a graph on *n* vertices with *m* edges. The SDP relaxation for Max-Cut starts out with a matrix variable of size *n* and *n* equations, while the evaluation of $\vartheta (G)$ requires a matrix of size $n+1$ and $n+m+1$ equations and in the computation of $t^{*}(G)$ there is a matrix of size $n+1$ and $2n + m$ equations. Hence the Max-Cut, stable set and coloring relaxation are contained in a $$\left( {\begin{array}{c}n\\ 2\end{array}}\right) $$, $$\left( {\begin{array}{c}n\\ 2\end{array}}\right) + n - m$$ and $$\left( {\begin{array}{c}n\\ 2\end{array}}\right) - m + 1$$ dimensional space, and it makes sense that Max-Cut has the most and coloring has the least violated ESCs, just as we see it in Table 1. Furthermore in the stable set and the coloring relaxation the additional row and column together with the positive semidefiniteness constraint effect all entries of *X*, even if they are not directly addressed by any constraint. Therefore it is plausible that the violations for the Max-Cut problem are much larger than those for the stable set and the coloring problem.

For our computations that means that there is the hope that fewer ESCs are necessary to tighten the basic relaxation. This intuition is indeed confirmed in our computational experiments in Sect. 7.

## Partial Lagrangian dual

We are interested in solving relaxations (4), (5) and (6) with a potentially large number of ESCs, where using interior point solvers is too time consuming. In this section we will first establish a unified formulation of the relaxations (4), (5) and (6). Then we will build the partial Lagrangian dual of this formulation, where only the ESCs are dualized.

In order to unify the notation for the three problems observe that the ESCs $X_{I}\in {{\,\mathrm{CUT}\,}}_{|I|}$, $X_{I}\in {{\,\mathrm{STAB}\,}}^{2}(G_{I})$ and $X_{I}\in {{\,\mathrm{COL}\,}}(G_{I})$ can be represented as

7 $$\begin{aligned} X_{I}= \sum _{i=1}^{t_{I}} \lambda _{i}C^{I}_{i},\quad \lambda \in {\varDelta }_{t_{I}}, \end{aligned}$$

where $C^{I}_{i}$ is the *i*-th cut, stable set or coloring matrix of the subgraph $G_{I}$ and $t_{I}$ is their total number.

A formal description of ESC in (7) requires some additional notation. First we introduce the projection ${{\mathcal {P}}}_I:{{\mathcal {S}}}_{n} \mapsto {{\mathcal {S}}}_{k_{I}}$, mapping *X* to the submatrix $X_{I}$. Second we define a map ${{\mathcal {A}}}_I:{{\mathcal {S}}}_{k_{I}} \mapsto {{\mathbb {R}}}^{t_{I}}$, such that its adjoint map ${{\mathcal {A}}}_I^{\top }:{{\mathbb {R}}}^{t_{I}} \mapsto {{\mathcal {S}}}_{k_{I}}$ is given by ${{\mathcal {A}}}_I^{\top }(\lambda )=\sum _{i=1}^{t_{I}}{\lambda _i C_i^{I}}$ and produces a linear combination of the cut, stable set or coloring matrices. Thus we can rewrite (7) as

8 $$\begin{aligned} {{\mathcal {A}}}_I^{\top }(\lambda _{I}) - {{\mathcal {P}}}_I(X) = 0, \quad \lambda _{I}\in {\varDelta }_{t_{I}}. \end{aligned}$$

The left-hand side of this matrix equality is a symmetric matrix, of which some entries (depending on which problem we consider) are zero for sure, so we do not have to include all $k_{I}\times k_{I}$ equality constraints into the SDP. Let $b_{I}$ be the number of equality constraints we have to include. Note that $$b_{I}= \left( {\begin{array}{c}k_{I}\\ 2\end{array}}\right) $$, $$b_{I}= \left( {\begin{array}{c}k_{I}\\ 2\end{array}}\right) + k_{I}- m_I$$ and $$b_{I}= \left( {\begin{array}{c}k_{I}\\ 2\end{array}}\right) - m_I$$ for the Max-Cut, stable set and coloring problem respectively, if $m_I$ denotes the number of edges of $G_{I}$. This is because in the case of the stable set problem we also have to include equations for the entries of the main diagonal contrary to Max-Cut and the coloring problem. Then we define a linear map ${{\mathcal {M}}}_I:{{\mathbb {R}}}^{b_{I}} \mapsto {{\mathcal {S}}}_{k_{I}}$ such that the adjoint operator ${{\mathcal {M}}}_I^{\top }:{{\mathcal {S}}}_{k_{I}} \mapsto {{\mathbb {R}}}^{b_{I}}$ extracts the $b_{I}$ positions, for which we have to include the equality constraints, into a vector. So we can rephrase (8) equivalently as

$$\begin{aligned} {{\mathcal {M}}}_I^{\top }({{\mathcal {A}}}_I^{\top }(\lambda _{I}) - {{\mathcal {P}}}_I(X)) = 0, \quad \lambda _{I}\in {\varDelta }_{t_{I}}, \end{aligned}$$

which are $b_{I}+1$ equalities and $t_{I}$ inequalities. In consequence all three relaxations (4), (5) and (6) have the generic form

9 $$\begin{aligned} z = \max \{ \langle C, {\widehat{X}} \rangle :~ {\widehat{X}} \in {{\mathcal {X}}},~ \lambda _{I}\in {\varDelta }_{t_{I}},~ {{\mathcal {M}}}_I^{\top }({{\mathcal {A}}}_I^{\top }(\lambda _{I}) - {{\mathcal {P}}}_I(X)) = 0 ~ \forall I \in J\}, \end{aligned}$$

where *C*, ${{\mathcal {X}}}$, ${{\mathcal {A}}}_I$, ${{\mathcal {M}}}_I$ and $b_{I}$ have to be defined in a problem specific way. Furthermore ${\widehat{X}} = X$ in the case of Max-Cut and stable set and $${\widehat{X}} = \left( \begin{array}{cc} t &{} \mathbb {1}_{}^{ {T} }\\ \mathbb {1}_{} &{} X \end{array}\right) $$ for coloring, but for the sake of understandability we will just use *X* in the following.

The key idea to get a handle on problem (9) is to consider the partial Lagrangian dual where the ESCs (without the constrains $\lambda _{I}\in {\varDelta }_{t_{I}}$) are dualized. We introduce a vector of multipliers $y_{I}$ of size $b_{I}$ for each *I* and collect them in $y= (y_{I})_{I\in J}$ and also collect $\lambda = (\lambda _{I})_{I\in J}$. The Lagrangian function becomes

$$\begin{aligned} {{\mathcal {L}}}(X,\lambda ,y) = \langle C, X \rangle + \sum _{I \in J}{\langle y_{I}, {{\mathcal {M}}}_I^{\top }({{\mathcal {A}}}_I^{\top }(\lambda _{I}) - {{\mathcal {P}}}_I(X)) \rangle } \end{aligned}$$

and standard duality arguments (Rockafellar [31, Corollary 37.3.2]) yield

10 $$\begin{aligned} z = \min _{y} \max _{\begin{array}{c} X \in {{\mathcal {X}}}\\ \lambda _{I}\in {\varDelta }_{t_{I}} \end{array}} {{\mathcal {L}}}(X,\lambda ,y). \end{aligned}$$

For a fixed set of multipliers $y$ the inner maximization becomes

$$\begin{aligned} \max _{\begin{array}{c} X \in {{\mathcal {X}}}\\ \lambda _{I}\in {\varDelta }_{t_{I}} \end{array}} \left\langle C - \sum _{I\in J}{{{\mathcal {P}}}_I^{\top }{{\mathcal {M}}}_I(y_{I})}, X \right\rangle + \sum _{I\in J}{\langle {{\mathcal {A}}}_I{{\mathcal {M}}}_I(y_{I}), \lambda _{I}\rangle }. \end{aligned}$$

This maximization is interesting in at least two aspects. First, it is separable in the sense that the first term depends only on *X* and the second one only on the separate $\lambda _{I}$. Moreover, if we denote the linear map ${{\mathcal {A}}}_I{{\mathcal {M}}}_I:{{\mathbb {R}}}^{b_{I}} \mapsto {{\mathbb {R}}}^{t_{I}}$ with the matrix ${{\mathcal {D}}}_I$, maximizing the summands of the second term is easy, because the feasible region is a simplex. Hence the explicit solution of maximizing a summand of the second term is

11 $$\begin{aligned} \max _{\lambda _{I}\in {\varDelta }_{t_{I}}}\langle {{\mathcal {D}}}_I(y_{I}), \lambda _{I}\rangle = \max _{1 \leqslant i \leqslant t_{I}}\left[ {{\mathcal {D}}}_I(y_{I}) \right] _{i}. \end{aligned}$$

In order to consider the first term in more detail, we define the following function. Let $b= \sum _{I\in J}b_{I}$ be the dimension of $y$. Then $h:{{\mathbb {R}}}^{b} \rightarrow {{\mathbb {R}}}$ is defined as

12 $$\begin{aligned} h(y)= \max _{X \in {{\mathcal {X}}}} \left\langle C - \sum _{I\in J}{{{\mathcal {P}}}_I^{\top }{{\mathcal {M}}}_I(y_{I})}, X \right\rangle = \left\langle C - \sum _{I\in J}{{{\mathcal {P}}}_I^{\top }{{\mathcal {M}}}_I(y_{I})}, X^{*} \right\rangle , \end{aligned}$$

where $X^{*}$ is a maximizer over the set ${{\mathcal {X}}}$ for *y* fixed. Note that $h(y)$ is convex but non-smooth, but (12) shows that

13 $$\begin{aligned} g_I= -{{\mathcal {M}}}_I^T {{\mathcal {P}}}_I(X^{*}) \end{aligned}$$

is a subgradient of *h* with respect to $y_{I}$.

With (11) and (12) we reformulate the partial Lagrangian dual (10) to

14 $$\begin{aligned} z = \min _{y} \left\{ h(y) + \sum _{I\in J}{\max _{1 \leqslant i \leqslant t_{I}}\left[ {{\mathcal {D}}}_I(y_{I}) \right] _i}\right\} . \end{aligned}$$

The dual formulation (14) of the original semidefinite relaxation (9) has the form of a convex minimization problem over the set of multipliers *y*. The evaluation of the function *h* at a given *y* requires solving a “simple” SDP, independent of the number of ESCs included in the relaxation.The function evaluation also provides a subgradient of *h* at *y*, given in (13). Hence we propose to use the *bundle method* from convex optimization to solve (14). The details are given in the subsequent section.

## Solving the partial Lagrangian dual

### The bundle method

The bundle method is a well established tool in convex optimization to minimize a non-smooth convex function. We refer to the recent monograph Bonnans, Gilbert, Lemaréchal and Sagastizábal [4] for a nice introduction. In our setting we want to use the bundle method in order to solve an SDP. Helmberg and Rendl [18] were the first to use a bundle method to solve SDPs in 2000. Later Fischer, Gruber, Rendl and Sotirov [10] and Rendl and Sotirov [29] used the bundle method for SDPs in order to get good relaxations for the Max-Cut and the equipartition problem and the quadratic assignment problem, respectively.

The bundle method setting described by Frangioni and Gorgone in [11], which is set up to handle $\max$ terms explicitly, is best suited for our purposes, so we apply it to our problem (14).

The bundle method is an iterative procedure. It maintains the *current center* ${\overline{y}}$, representing the current estimate of the optimal solution, and the set ${\mathcal {B}}= \{ (y_{1}, h_{1},g_{1},X_{1}), \dots , (y_{r}, h_{r},g_{r},X_{r}) \}$, which is called *bundle*, throughout the iterations. Here $y_1, \ldots , y_r$ are the points which we use to set up our subgradient model. Moreover $h_j = h(y_j)$, $g_j$ is a subgradient of *h* at $y_j$ and $X_j$ is a maximizer of *h* at $y_j$ as in (12).

At the start we select $y_1={\overline{y}}=0$ and evaluate *h* at ${\overline{y}}$, which yields the bundle ${\mathcal {B}}=\{(y_1, h_1,g_1,X_1)\}$. A general iteration consists of first determining the new *trial point*, then evaluating the function at this new point, and finally updating the bundle ${\mathcal {B}}$. In the literature evaluating the function is referred to as calling the *oracle*. The subgradient information of the bundle ${\mathcal {B}}$ translates into the subgradient model

$$\begin{aligned} h(y) \geqslant h_{j} + \langle g_{j},y-y_{j}\rangle \text { for all } j = 1, \dots , r. \end{aligned}$$

It is common to introduce

$$\begin{aligned} e_{j} = h({\overline{y}}) - h_{j} - \langle g_{j},{\overline{y}}-y_{j}\rangle \text { for } j = 1, \dots , r\end{aligned}$$

and to define $e = (e_j)_{j=1,\dots ,r}$. With ${\overline{h}}= h({\overline{y}})$ the subgradient model becomes

15 $$\begin{aligned} h(y) \geqslant \max _{1\leqslant j \leqslant r} \left\{ {\overline{h}}- e_{j} + \langle g_{j},y-{\overline{y}}\rangle \right\} . \end{aligned}$$

The right-hand side above is convex, piecewise linear and minorizes *h*. In each iteration of the bundle method we minimize the right-hand side of (15) instead of *h*, but ensure that we do not move too far from ${\overline{y}}$ by adding a penalty term of the form $\frac{1}{2}\mu \left\Vert y-{\overline{y}} \right\Vert _2^{2}$ for a parameter $\mu \in {{\mathbb {R}}}_{+}$ to the objective function. We introduce auxiliary variables $w \in {{\mathbb {R}}}$ and $v_{I}\in {{\mathbb {R}}}$ for all $I \in J$ to model the maximum terms. With $q= |J|$ and $v = (v_{I})_{I \in J} \in {{\mathbb {R}}}^q$ we end up with16This is a convex quadratic problem in $1+q+b$ variables with $r+\sum _{I \in J}t_{I}$ linear inequality constraints which is often referred to as the *bundle master problem*. Its solution $({\widetilde{y}},{\widetilde{w}},{\widetilde{v}})$ provides the new trial point ${\widetilde{y}}$. In the following section we will discuss computational issues and present a practically efficient approach starting with its dual, see below.

The second step in each bundle iteration is to evaluate the function *h* at ${\widetilde{y}}$ which means solving the basic SDP relaxation as introduced in Sect. 2 with a modified objective function. In the case of Max-Cut this function evaluation can be done very quickly (solve an SDP with *n* simple equations). For the stable set and the coloring problem the resulting SDP is computationally more demanding, as there are also equations for each edge in the graph. The bundle iteration is finished by deciding whether ${\widetilde{y}}$ becomes the new center (serious step, roughly speaking if the increase of the objective function is good enough) or not (null step). In either case the new point is included in the bundle, some other elements of the bundle are possibly removed, the bundle parameter $\mu$ is updated and a new iteration starts.

### The dual of the bundle master problem

In the bundle method it is commonly proposed to solve the dual problem of (16), hence next we derive the dual of (16). Towards this end we collect the subgradients $g_{i}$ in the matrix ${{\mathcal {G}}}=(g_{1}, \ldots , g_{r})$. It will be notationally convenient to partition the matrix ${{\mathcal {G}}}$ into blocks of rows corresponding to the subsets $I \in J$, namely ${{\mathcal {G}}}= ({{\mathcal {G}}}_I)_{ I \in J }$ where each ${{\mathcal {G}}}_I$ has *r* columns and $b_{I}$ rows. Furthermore we make the subgradient model and maximum term constraints more compact by reformulating them to $w \mathbb {1}_{} \geqslant {\overline{h}}\mathbb {1}_{} - e + \sum _{I \in J} {{\mathcal {G}}}_I^{\top }(y_{I}-{\overline{y}}_{I})$ and $v_{I}\mathbb {1}_{} \geqslant {{\mathcal {D}}}_I(y_{I})$.

We denote by $\alpha \in {{\mathbb {R}}}^{r}$ the dual variables to the subgradient model constraints and with $\beta _{I}\in {{\mathbb {R}}}^{t_{I}}$ the dual variables of the constraints involving $v_{I}$ for the maximum terms. Furthermore we define $\beta = (\beta _{I})_{I \in J}$ as the collection of all $\beta _{I}$. Hence we obtain the Lagrangian function

$$\begin{aligned} {{\mathcal {L}}}(y,w,v,\alpha ,\beta ) = w&+ \sum _{I \in J}v_{I}+ \frac{1}{2}\mu \sum _{I \in J}\left\Vert y_{I}-{\overline{y}}_{I} \right\Vert _{2}^{2} \\&+ \left\langle \alpha , {\overline{h}}\mathbb {1}_{} -e - w\mathbb {1}_{} \right\rangle + \sum _{I \in J} \left\langle \alpha , {{\mathcal {G}}}_I^{\top }(y_{I}-{\overline{y}}_{I}) \right\rangle \\&+ \sum _{I \in J} \left\langle \beta _{I}, {{\mathcal {D}}}_I( y_{I}) - v_{I}\mathbb {1}_{} \right\rangle . \end{aligned}$$

After exchanging $\min$ and $\max$ by using strong duality the dual of (16) becomes

$$\begin{aligned} \max _{\begin{array}{c} \alpha \geqslant 0\\ \beta \geqslant 0 \end{array}} \text { } \min _{y,w,v} {{\mathcal {L}}}(y,w,v,\alpha ,\beta ). \end{aligned}$$

Since $\nabla _{w}{{\mathcal {L}}}=0$,  $\nabla _{v_{I}}{{\mathcal {L}}}= 0$, and $\nabla _{y_{I}} {{\mathcal {L}}}= 0$ has to hold for all $I \in J$ at the dual optimum, we get $\alpha \in {\varDelta }_{r}$, $\beta _{I}\in {\varDelta }_{t_{I}}$ and

17 $$\begin{aligned} y_{I}= {\overline{y}}_{I}- \frac{1}{\mu }\left( {{\mathcal {G}}}_I(\alpha ) + {{\mathcal {D}}}_I^{\top }(\beta _{I})\right) . \end{aligned}$$

In consequence the dual of (16) simplifies to

18 $$\begin{aligned} \max _{\begin{array}{c} \alpha \in {\varDelta }_{r}\\ \beta _{I}\in {\varDelta }_{t_{I}} \end{array}} {\overline{h}}- e^{T} \alpha + \sum _{ I \in J }\left\langle {{\mathcal {D}}}_I({\overline{y}}_{I}) , \beta _{I}\right\rangle - \frac{1}{2\mu }\sum _{I \in J} \left\Vert {{\mathcal {G}}}_I(\alpha ) + {{\mathcal {D}}}_I^{\top }(\beta _{I}) \right\Vert ^{2}_{2}. \end{aligned}$$

This is a convex quadratic problem with $r + \sum _{I\in J} t_{I}$ variables and $1+q$ simple equality constraints, asking that the respective block of variables adds up to one. Now instead of solving (16) within the bundle method directly, we solve its dual (18) to get the multipliers $\alpha$ and $\beta$ and recover ${\widetilde{y}}$ using (17).

### Our bundle method

So far we have sketched how to use our bundle method in order to obtain a solution *y* of (14), but actually we are interested in a solution *X* of (9). One can use the bundle ${\mathcal {B}}= \{ (y_{1}, h_{1},g_{1},X_{1}), \dots , (y_{r}, h_{r},g_{r},X_{r}) \}$, which is updated in each iteration, in order to obtain a good approximate solution for *X*. In particular it follows from the convergence theory of the bundle method that under mild conditions

19 $$\begin{aligned} X = \sum _{j=1}^{r} \alpha _j X_j \quad \text {and}\quad \lambda _{I}= \beta _{I}\end{aligned}$$

converges to the optimal values of *X* and $\lambda _{I}$ of (9), see for example Robinson [30] for the general theory and Gaar [12] for the convergence in our particular setting.

We are now able to present our version of the bundle method. Note that there is no need of keeping $y_j$ in the bundle explicitly by computing and updating *e* in a proper way, so we drop $y_j$ from the bundle ${\mathcal {B}}$. Algorithm 1 summarizes the main computational steps of our bundle method to get an approximate optimal solutions of (9) and (14).
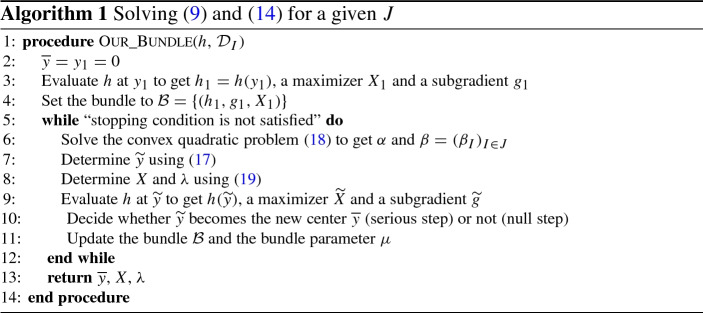


The generic description of our bundle method in Algorithm 1 leaves some flexibility to the user. We will present implementation details in Sect. 6.3.

## The computation of ESCs based bounds

### The overall algorithm

The goal of this paper is to get good bounds on the optimal Max-Cut value $z_{mc}$, the stability number $\alpha (G)$ and the chromatic number $\chi (G)$ by including ESCs into the basic SDP relaxations (1), (2) and (3) in order to improve the bounds from the basic SDP relaxations. We will call bounds obtained in this way *exact subgraph bounds (ESB)*. In other words ESBs are attained by solving (4), (5) and (6) or, in the generic form, by solving (9).

Up to now we have concentrated on the most subtle part of retrieving good ESBs, which consists in solving the SDP relaxation (9) with a given set *J* of ESCs. Our ultimate goal however is to reach ESBs where all ESCs of order *k* are (nearly) satisfied for small values of *k* like $k\leqslant 7$.

We propose to reach this goal by proceeding iteratively. Starting with $k=3$ in the Max-Cut case (as there are no violated ESCs of order 2) and $k=2$ in the other cases we search for violated ESCs of order *k* and include only the most violated ESCs that we find into *J*. After solving the SDP (9), we follow an extreme strategy and remove any ESC that has become inactive. As we typically still find further badly violated ESCs this allows us a quick exploration of the entire space of ESCs. Once we do not find ESCs of order *k* with significant violation, we increase *k* and continue. We call each such iteration a *cycle*.

In each cycle so we keep some information, such as the current dual variables $y_i$ and the bundle ${\mathcal {B}}$, appropriately modified to reflect possibly deleted and added new constraints. In particular we delete from all $y_i$ the positions corresponding to deleted ESCs, extend all $y_i$ with zeros for the newly added ESCs and deduce the update of all other variables. This choice allows us to reuse the bundle ${\mathcal {B}}$. Our procedure to compute ESBs is sketched in Algorithm 2.
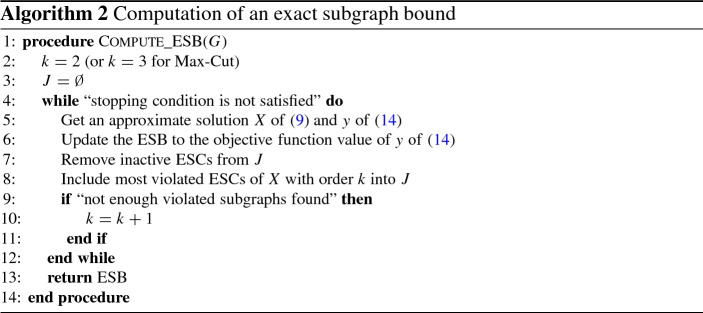


**Fig. 4:**
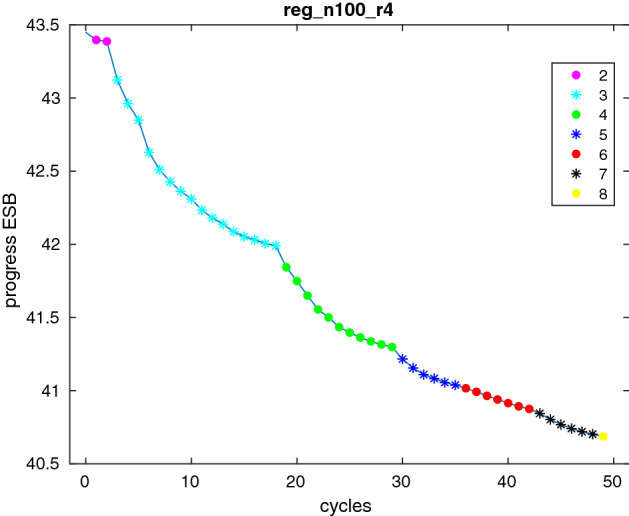
Progress of the ESB over 50 cycles for one instance of Table 4

The typical behavior over a set of cycles for one stable set instance can be seen in Fig. 4. After only a few cycles with $k=2$ we move to $k=3$. Here it takes 16 cycles to reach a point with all ESCs nearly satisfied. The Figure clearly shows a continuing improvement of the ESB over the cycles.

Note that the ESB computed in Algorithm 2 is indeed a valid bound, because any *y* is feasible for (14) and hence its dual objective function value is a valid bound on the primal optimal objective function value (9), which in turn is a valid bound on the optimal objective function value of the combinatorial optimization problem. Hence it is not necessary to solve (9) and (14) to optimality to obtain valid bounds. Of course we want to use our bundle method, Algorithm 1, in order to obtain the approximate solutions in line 5 of Algorithm 2.

### Finding violated exact subgraph constraints

The key ingredients of Algorithm 2 are on the one hand Algorithm 1, which was detailed in Sect. 5, and on the other hand the update of the set *J*.

The crucial point in order to do so is to find violated ESCs. Let $G_{I}$ be a subgraph of oder $k_{I}$ of *G* and $X^*$ be the current solution of (9) and let *U* be an arbitrary $k_{I}\times k_{I}$ matrix. Clearly ${{\,\mathrm{CUT}\,}}_{k_{I}}$, ${{\,\mathrm{STAB}\,}}^2(G_{I})$ and ${{\,\mathrm{COL}\,}}(G_{I})$ are bounded polytopes, hence the inner product of any element of these polytopes with *U* is contained in a certain interval. Thus finding *I* such that the inner product of *U* with the submatrix $X_{I}^*$ of $X^*$ is minimum identifies a potentially violated subgraph.

This minimization may be recast as a quadratic assignment problem consisting of the data matrices $X^{*}$ and the matrix *U* embedded in an $n \times n$ matrix. We repeatedly use a local search heuristic for different fixed *U* in order to obtain potentially violated subgraphs. Then we compute the projection distances of $X_{I}^*$ to the corresponding polytope for all these subgraphs $G_{I}$ and include those into *J* which have the largest projection distances and hence are violated most.

Possible choices for *U* make use of hyperplanes for the respective target polytope, but other choices are possible. In our computations we use a collection of different matrices for *U*, for example matrices that induce facets of the corresponding polytope (if their computation for a particular $k_{I}$ is possible easily, which is the case for $k_{I}\leqslant 6$), extreme copositive matrices with $\{0,1,-1\}$ entries and random matrices. For each cycle we use at most 50 different matrices *U*.

### Details of the bundle implementation

We now briefly discuss some details of our implementation of Algorithm 1 when used in line 5 of Algorithm 2. First of all one needs to decide on a stopping condition. Ideally we would stop, once a subgradient equal to zero is found. In our case, we either stop once the norm of the new subgradient is small enough (in the case of Max-Cut), or once the difference of the value of the function at the current center point and the value of the subgradient model of the function at the new trial point is smaller than some tolerance (as it is done in [4], the tolerance is 0.005 in our implementations) or once we reach a maximum number of iterations (30 in our implementations). The third condition is motivated by the fact that we typically will continue adding new violated ESCs, so there is no real need to get the exact minimum of (14). Note however that it is important to come close to the optimal solution, because otherwise the resulting *X* does not have a high enough precision in order to be useful for finding new violated subgraphs.

For updating the bundle we always add the new trial point to the bundle, but remove subgradients from the bundle that have become inactive. This extreme choice of updating the bundle led to the best performance in our computational experiments. In order to update the bundle parameter $\mu$ we use a modification of an update proposed by Kiwiel [21]. We perform a serious step whenever the improvement of the objective function value of the new trial point is at leas a certain fraction of the expected improvement. This is a standard criterion, see for example [19]. We solve the bundle master problem as a rotated second-order cone program (see [2] for more details) with MOSEK.

## Computational results

### Bundle approach versus interior point methods

We start our computational investigation with a comparison of our bundle method with an interior point method in order to solve (9). In our overall Algorithm 2 presented in Sect. 6 this has to be done in each cycle, so we are highly interested in fast running times.

From a theoretical point of view it is clear which method will win this competition: Assume we include $q = 1000$ ESCs (so $q = |J|$) for subgraphs of order $k_{I}= 5$ in (9) for the stable set problem. Then we have $t_{I}\leqslant 2^5 = 32$ stable set matrices that potentially span ${{\,\mathrm{STAB}\,}}^2(G_{I})$, and up to $$b_{I}\leqslant \left( {\begin{array}{c}k_{I}\\ 2\end{array}}\right) + k_{I}= 15$$ equality and one inequality constraint for each ESC. In total we have up to 32000 variables that have to fulfill up to 16000 constraints in (9)—additionally to the variables and constraints of the basic SDP relaxation (2). It is clear that the number of constraints will be a challenge for an interior point solver. In particular an interior point solver has to solve this SDP with a large number of constraints at once, whereas our bundle method in Algorithm 1 “only” has to solve the basic SDP relaxation and the bundle master problem over several iterations. Therefore, we expect the bundle method to be the clear winner in this competition and refrain from a large scale comparison.

Instead, we compare the two methods only on some instances to confirm our theoretical inspection. In Table 2 we list the results for one Max-Cut and one stable set instance, both are taken from the Erdős-Rényi model *G*(*n*, *p*). We vary the number of included ESCs for subgraphs of order 3, 4 and 5, so we solve (4) and (5) for different *J*. We choose *J* such that the total number of equality constraints induced by the convex hull formulation of the ESCs *b* ranges between 6000 and 15000. On the one hand we solve the instances with two interior point solvers, namely MOSEK and SDPT3 [33, 34] and list the running times in seconds. On the other hand we use our bundle method. In our context we are mostly interested to improve the upper bounds quickly, so we do not run Algorithm 1 until we reach a minimizer, but stop after 30 iterations. We list the running time for the oracle, i.e. the sum of the solution times of the basic SDP relaxation, and the overall running times. Additionally we present how much $\%$ of the MOSEK running time the bundle method needs and how close the solution found by the bundle method is to solution of MOSEK in $\%$ ($100\%$ means the solutions coincide).

In Table 2 one sees that the running times decrease drastically if we use the bundle method compared to interior point solvers. For $b\approx 15000$ it takes the bundle method only around $8\%$ of the MOSEK running time to get as close as $95\%$ to the optimal value, which is sufficient for our purposes. One sees that our bundle method scales much better for increasing |*J*|, so for an increasing number of ESCs. Furthermore MATLAB requires 12 Gigabyte of memory with interior point solvers for $b=15000$, showing also memory limitations.

**Table 2:** Running times for one Max-Cut and one stable set instance with different sets of ESCs, where the graphs of order $n=100$ are from the Erdős-Rényi model

					Interior point		Algorithm 1			
	#ESC of order				Time (s)		Time (s)		$\%$ of MOSEK	
	3	4	5	*b*	MOSEK	SDPT3	Oracle	Overall	Time	Value
MC	2000		0	6000	18.37	49.22	1.01	6.05	32.93	97.20
	2000		300	9000	55.24	134.78	1.18	9.33	16.90	95.02
	4000		0	12000	104.56	289.78	1.71	11.13	10.64	93.66
	3000		600	15000	184.43	525.85	1.56	14.83	8.04	94.54
SS	1050	0	0	5914	23.54	79.25	7.86	10.65	45.22	98.25
	1050	212	63	8719	50.11	174.33	10.61	16.52	32.96	97.89
	2100	0	0	11780	126.40	388.07	7.43	12.27	9.71	93.65
	1575	318	212	14653	241.29	648.83	10.79	20.21	8.38	94.44

To summarize our small computational investigation confirms our intuition that the bundle method is much better suited for our purposes.

We want to point out that the number of bundle iterations can be increased in order to get closer to the optimum. For the larger instances in Table 2 this will still result in significantly shorter running times.

Note that the bundle method has another advantage: A warm start with the bundle ${\mathcal {B}}$ and the solution ${\overline{y}}$ of the previous iteration in line 5 of Algorithm 2 is possible. Since many ESCs remain the same in *J* the problem to solve in line 5 does not change too much and a warm start can be very beneficial.

As a last remark we want to draw the attention to the running times for the oracle in Table 2. For the stable set problem the oracle needs over half of the running time, whereas in the Max-Cut problem the oracle evaluation is much faster. This is due to the fact that the basic SDP relaxation is a simpler SDP for the Max-Cut problem.

In the following we present several computational results for obtained ESB by using the bundle method. Note that we refrain from comparing the running times of our bundle method with the running time of interior point methods, because interior point methods would reach their limit very soon.

### The stable set and the coloring problem

In this section we will extend the computational results from [13] for the stable set and the coloring problem. The computational investigations show that (i) the ESB obtained by including ESCs of fixed order $k_{I}$ improve for increasing $k_{I}$ and (ii) after including several ESCs for subgraphs of order $k_{I}$ the maximum projection distance of the violated subgraphs found decreases drastically.

We extend these computational results by deriving one final ESB for several instances with Algorithm 2. We stop as soon as we have performed 50 cycles and only include subgraphs of order $k \leqslant 8$. We add at most 100 ESCs in each cycle and warmstart the bundle with the information of the previous cycle. We already saw the typical behavior of the ESB over the cycles in Fig. 4.

**Table 3:** Stable set results for torus graphs

*d*	*n*	*m*	$\vartheta (G)$	Final ESB	$\alpha (G)$	Time oracle	Time other
5	25	50	11.180	10.002	10	14.45	18.58
7	49	98	23.224	21.009	21	95.06	31.96
9	81	162	39.241	36.021	36	277.98	66.37
11	121	242	59.249	55.066	55	728.78	122.46
13	169	338	83.254	79.084	78	859.73	170.85
15	225	450	111.257	106.287	105	1390.52	224.97
17	289	578	143.259	136.821	135	3123.41	314.29

As a first structural easy class of graphs we consider two-dimensional torus graphs which are constructed as follows. For given *d*, the graph $T_d$ has $d^2$ vertices which we label by (*i*, *j*) for $i,j \in \{1, \ldots ,d\}$. The vertical edges join vertices with neighboring *i* indices (and *j* fixed), yielding edges $\{(i,j), (i+1,j)\}$ modulo *d*, and similarly the horizontal edges join vertices with *i* fixed $\{(i,j), (i,j+1)\}$ modulo *d*. So there is a total of $n=d^2$ vertices and $m=2n$ edges. It is not hard to verify that in case of odd $d=2t+1$, we get $\alpha (T_d)=t(2t+1)$ and if $d=2t$ we have $\alpha (T_d) = 2t^2$. The even case is not interesting, as $\vartheta (T_d)= \alpha (T_d)$. For *d* odd we summarize some computational results in Table 3. We observe that for these graphs our ESB is substantially better than $\vartheta (G)$ and we close the integer gap for all instances with $n \leqslant 121$.

When considering the running times observe that the majority of the running time (given in seconds in Table 3) is used for the oracle, because the SDP to evaluate $\vartheta (G)$ given in (2) with a slightly modified objective function is nontrivial. We tried several solver to solve this SDP, among them the interior point solver MOSEK [26], and solvers based on alternating direction method of multipliers as DADAL [7] and SDPNAL+ [35]. Both these solvers show very good results on computing $\vartheta (G)$, but as soon as the objective function slightly changes they do not perform well anymore. Hence it will be future research to develop an SDP solver dedicated to these kind of instances. Note that the running time in order to perform Algorithm 2 is not very high and in particular only increases mildly for larger instances.

As a second class of problems we consider random near-*r*-regular graphs, which we generate as follows. We select a perfect matching on *nr* vertices and then we identify consecutive groups of *r* vertices into a single vertex. This yields a regular multigraph on *n* vertices. We remove loops and multiple edges resulting in a near-regular graph. In Tables 4 and 5 we provide results for random graphs. We compare near-regular graphs with random graphs from the Erdős-Rényi model where the density *p* is chosen so that the number of edges roughly matches those of the regular graphs. We compute our ESB and use a heuristic to compute large stable sets. In the results the gap between $\vartheta (G)$ and $\alpha (G)$ seems to be bigger for regular graphs, but we see in both cases that the ESB reduce the gap between $\vartheta (G)$ and the cardinality of the largest stable set found in a nontrivial way. Concerning running times we observe the same behavior as before.

**Table 4:** Stable set results for near-regular graphs

graph	*n*	*r*	*m*	$\vartheta (G)$	Final ESB	$\alpha (G)\geqslant$	Time oracle	Time other
reg_n100_r4	100	4	195	43.449	40.687	40	1020.60	143.12
reg_n100_r6	100	6	294	37.815	35.246	34	935.69	125.93
reg_n100_r8	100	8	377	34.480	32.190	31	939.27	127.46
reg_n200_r4	200	4	400	87.759	83.732	80	1278.61	158.67
reg_n200_r6	200	6	593	79.276	75.555	68	1362.04	160.90
reg_n200_r8	200	8	792	70.790	67.785	60	1751.27	192.74
reg_n200_r10	200	10	980	66.418	62.894	57	2356.94	199.12

**Table 5:** Stable set results for graphs from the Erdős-Rényi model *G*(*n*, *p*)

graph	*n*	*m*	$\vartheta (G)$	Final ESB	$\alpha (G)\geqslant$	Time oracle	Time other
rand_n100_p004	100	212	46.067	45.021	45	338.77	93.34
rand_n100_p006	100	303	40.361	38.439	38	769.80	117.33
rand_n100_p008	100	443	34.847	32.579	32	1126.16	135.89
rand_n100_p010	100	489	34.020	32.191	32	1004.05	134.39
rand_n200_p002	200	407	95.778	95.032	95	679.90	155.80
rand_n200_p003	200	631	83.662	81.224	80	1672.94	193.75
rand_n200_p004	200	816	73.908	70.839	67	2035.58	191.42
rand_n200_p005	200	991	69.039	66.091	62	2195.78	215.50

As a last experiment for the stable set problem in Table 6 we consider instances from the literature, taken mostly from the DIMACS challenge [9]. On some instances there is hardly any improvement of the bound, while other instances are solved to optimality. It requires future research to get a better understanding for the fluctuation in quality on these instances, but for almost all instances the bound improves by at least one integer value.

The computation times for these instances range from 200 to 500 s for the smaller instances ($n \leqslant 125$) to several hours for the biggest graphs. As in the instances before a faster oracle would improve the running times substantially.

Note that in our computations we aim for getting as good bounds as possible. If one wants to use the bounds in a branch-and-bound setting, a much more aggressive strategy with increasing $k_{I}$ faster and stopping as soon as we do not expect to reach the next integer value is favorable.

**Table 6:** Tighten $\vartheta (G)$ towards $\alpha (G)$

Graph	*n*	*m*	$\vartheta (G)$	Final ESB	$\alpha (G)\geqslant$	Time oracle	Time other
Circulant47_030	47	282	14.302	13.019	13	193.31	39.95
PaleyGraph61	61	915	7.810	7.027	5	319.37	51.79
hamming6_4	64	1312	5.333	4.005	4	71.37	13.93
spin5	125	375	55.902	50.004	50	342.20	71.68
keller4	171	5100	14.012	13.505	11	1923.60	62.34
sanr200_0_9	200	2037	49.274	47.614	42	5171.69	253.94
c_fat200_5	200	11427	60.345	58.001	58	3601.56	85.42
p_hat300_1	300	8773	160.345	160.342	158	5106.69	112.59
p_hat300_2	300	6092	160.345	158.000	158	31228.51	187.41
p_hat300_3	300	3072	160.345	158.000	158	19563.05	252.68

**Table 7:** Tighten $t^{*}(G)$ towards $\chi (G)$

Graph	*n*	*m*	$t^{*}(G)$	Final ESB	$\chi (G)\leqslant$	Time overall
myciel3	11	20	2.400	3.276	4	236.95
myciel4	23	71	2.529	3.505	5	1422.71
myciel5	47	236	2.639	3.510	6	4240.61
mug88_1	88	146	3.000	3.022	4	4709.40
1_FullIns_4	93	593	3.124	3.939	5	7219.83
myciel6	95	755	2.734	3.534	7	1540.82
myciel7	191	2360	2.820	3.582	8	2295.24
2_FullIns_4	212	1621	4.056	4.700	6	10106.29
flat300_26_0	300	21633	16.998	17.121	26	7535.75

Results for a selection of coloring instances from [27] are provided in Table 7. As in the case of the stable set problem we use Algorithm 2 to obtain ESBs. We include at most 100 ESCs in each cycle, only include ESCs for subgraphs of order $k \leqslant 8$ and perform at most 25 cycles. The results are similar in quality to those for stable set from Table 6, so for the most instances we are able to obtain bounds, which are one integer value better than the original bounds from $t^{*}(G)$. The large running times are due to the difficult basic SDP relaxation (3).

### The Max-Cut problem

Finally we are ready to present computational results for the Max-Cut problem. It is well known that in the basic SDP relaxation of Max-Cut (1) all ESCs of order 3 can equivalently be represented by the metric polytope [23]. Optimizing over it gives the exact solution to Max-Cut on graphs not contractible to $K_5$, in particular on planar graphs. It is also well known that optimizing over the metric polytope may lead to rather weak relaxations for general graphs. In contrast, the simple SDP relaxation (1) provides an upper bound at most 14% above the optimal value of Max-Cut for graphs with nonnegative edge weights, see [14].

In our computational experiments with Max-Cut we noted that the number of ESCs necessary to insure that all ESCs for a given value *k* are satisfied can be quite large (see Sect. 3.2), even for small values of *n*, such as $n=100$. We therefore simplify the ESC relaxation further. If a subgraph $G_{I}$ violates the ESC, then instead of asking that $X_I \in CUT_k$, we generate a single linear inequality separating $X_I$ from $CUT_k$ and include it instead of the ESC. This weakens the relaxation, but also reduces the computational effort, so that the total number of ESCs in the model may be quite large, and we can still compute the ESB. The computational effort is quite moderate, requiring no more than about 120 s for each of the instances.

**Table 8:** Max-Cut results for graphs from the Erdős-Rényi model *G*(*n*, *p*)

*n*	*p*	3	7	$z_{mc}$	#ESC
	0.10	0.70	0.07	118	2687
100	0.25	3.77	0.86	180	3705
	0.50	5.20	2.53	246	3521
	0.10	7.50	4.78	184	4755
150	0.25	7.39	5.02	310	4779
	0.50	9.71	7.51	459	4605

We first consider random graphs on *n* vertices from the Erdős-Rényi model *G*(*n*, *p*). Each edge is then assigned the weight 1 or $-1$ (each with probability 1/2). In Table 8 we report our computational results for $n\in \{100, 150 \}$ and $p \in \{0.1, 0.25, 0.5 \}$. We compare the ESB with $k=3$ (column labeled 3) to the ESB with $k = 7$ (column labeled 7). The column labeled 3 provides the deviation (in %) of the ESB with $k=3$ from $z_{mc}$. Thus if *p* is the value in the column labeled 3, then the ESB is equal to $(1 + p/100)z_{mc}$. The column labeled 7 is to be understood in an analogous way for $k=7$. In all cases we note a substantial gap reduction going from $k=3$ to $k=7$. The last column contains the number of ESCs at termination. It ranges from about 3000 for $n=100$ to about 4500 for $n=150$ and justifies our strategy to represent each ESC through a single cutting plane.

Next we consider graphs from the Beasley collection [3] with $n=250$. Rendl, Rinaldi and Wiegele [28] used 10 of these instances in a branch-and-bound setting. The “hardest” instance 250-08 reported in [28] resulted in 4553 nodes in the branch-and-bound tree and took several days of computation time. All the other 9 instances from this collection resulted in branch-and-bound trees having between 17 and 223 nodes with computation times in the order of hours, see Table 6 from [28]. We recomputed the root bound for all these instances and present our root gap in Table 9. We find it remarkable that our new bounding procedure is strong enough to prove optimality for all these instances right at the root node with the exception of problem 250-08. For this problem the gap at the root node was 2.19%. We recomputed the root bound in our setting and came up with a root gap of only 0.5%, thus reducing the gap by 75%.

**Table 9:** Max-Cut results for graphs from the OR library

Graph	Opt. cut	# Branch-and-bound nodes [28]	Root gap [28]	Our root gap
250-01	45607	37	0.44	*
250-02	44810	19	0.56	*
250-03	49037	19	0.14	*
250-04	41274	17	0.39	*
250-05	47961	21	0.35	*
250-06	41014	223	1.03	*
250-07	46757	37	0.44	*
250-08	35726	4553	2.19	0.5
250-09	48916	47	0.78	*
250-10	40442	63	0.62	*

**Table 10:** Max-Cut results for Chimera graphs with $n=512$

Graph	Final ESB	Best found cut	#ESC
chimera-1	434.38	433	22275
chimera-2	452.69	451	24707
chimera-3	447.35	447	22390
chimera-4	439.90	439	20748
chimera-5	441.66	440	22838

As a final experiment we consider Max-Cut instances on *Chimera graphs*. This class of graphs has found increased interest in connection with quantum annealing, see [6] for further details. In Table 10 we provide computational results with such graphs on $n=512$ vertices. We compute our ESB and also use a heuristic to find a good cut. It turns out that our bounding approach works nicely on these graphs, leading to provably optimal solutions in 2 out of 5 instances and the smallest possible positive gap (of 1) in the remaining cases. The computation times for each of these (big) instances range from 700 to 900 s, which we consider remarkable when dealing with more than 20000 ESCs.

We conclude that for Max-Cut our ESB constitute a substantial improvement compared to the previously used strongest bounds based on SDP with triangle inequalities [28]. These correspond to the column labeled 3 in Table 8.

## Conclusions and future work

Summarizing, we offer the following conclusions from the computational results. Our computational approach based on the partial Lagrangian dual is very efficient in handling also a large number of ESCs. The dual function evaluation separates the SDP part from the ESCs and therefore opens the way for large-scale computations. The minimization of the dual function is carried out as a convex quadratic optimization problem without any SDP constraints, and therefore is also suitable for a large number of ESCs.

Our computational results for stable set and coloring confirm the theoretical hardness results for these problems. Including ESCs of rather small size ($k \leqslant 8$) yields a noticeable improvement of the bounds.

The limiting factor for stable set instances is the solution time of the oracle. Hence it is desirable to have a fast solver for these kind of instances.

On the practical side we consider the cutting plane weakening of the ESCs for Max-Cut a promising new way to tighten bounds for this problem.

It will be a future project to explore these bounds in a branch-and-bound setting in order to solve Max-Cut, stable set and coloring instances to optimality.

## References

[1] Adams E, Anjos MF, Rendl F, Wiegele A (2015). A Hierarchy of subgraph projection-based semidefinite relaxations for some NP-hard graph optimization problems. INFOR Inf. Syst. Oper. Res..

[2] Alizadeh, F., Goldfarb, D.: Second-order cone programming. Math. Program. 95(1, Ser. B), 3–51, iSMP 2000, Part 3. Atlanta, GA (2003)

[3] Biq Mac Library: http://biqmac.aau.at/. Last accessed 15 May 2019

[4] Bonnans JF, Gilbert JC, Lemaréchal C, Sagastizábal CA (2006). Numerical Optimization: Theoretical and Practical Aspects.

[5] Boros E, Crama Y, Hammer PL (1990). Upper-bounds for quadratic $$0$$-$$1$$ maximization. Oper. Res. Lett..

[6] Dash S, Puget JF (2015). On quadratic unconstrained binary optimization problems defined on Chimera graphs. Optima.

[7] De Santis, M., Rendl, F., Wiegele, A.: Using a Factored Dual in Augmented Lagrangian Methods for Semidefinite Programming. ArXiv e-prints (Oct 2017)

[8] Delorme C, Poljak S (1993). Laplacian eigenvalues and the maximum cut problem. Math. Progr.

[9] DIMACS Implementation Challenges: http://dimacs.rutgers.edu/Challenges/ (1992). Last accessed 15 May 2019

[10] Fischer I, Gruber G, Rendl F, Sotirov R (2006). Computational experience with a bundle approach for semidefinite cutting plane relaxations of Max-Cut and equipartition. Math. Program..

[11] Frangioni A, Gorgone E (2014). Bundle methods for sum-functions with “easy” components: applications to multicommodity network design. Math. Program..

[12] Gaar, E.: Efficient Implementation of SDP Relaxations for the Stable Set Problem. Ph.D. thesis, Alpen-Adria-Universität Klagenfurt (2018)

[13] Gaar E, Rendl F, Lodi A, Nagarajan V (2019). A bundle approach for SDPs with exact subgraph constraints. Integer Programming and Combinatorial Optimization.

[14] Goemans MX, Williamson DP (1995). Improved approximation algorithms for maximum cut and satisfiability problems using semidefinite programming. J. Assoc. Comput. Mach..

[15] Grötschel M, Lovász L, Schrijver A (1988). Geometric Algorithms and Combinatorial Optimization, Algorithms and Combinatorics: Study and Research Texts.

[16] Guruswami V, Khanna S (2004). On the hardness of 4-coloring a 3-colorable graph. SIAM J. Discrete Math..

[17] Håstad J (1999). Clique is hard to approximate within $$n^{1-\epsilon }$$. Acta Math..

[18] Helmberg C, Rendl F (2000). A spectral bundle method for semidefinite programming. SIAM J. Optim..

[19] Hiriart-Urruty JB, Lemaréchal C (1993). Convex Analysis and Minimization Algorithms II: Advanced Theory and Bundle Methods.

[20] Khot, S.: Improved inapproximability results for MaxClique, chromatic number and approximate graph coloring. In: 42nd IEEE Symposium on Foundations of Computer Science (Las Vegas, NV, 2001), pp. 600–609. IEEE Computer Society, Los Alamitos, CA (2001)

[21] Kiwiel KC (1990). Proximity control in bundle methods for convex nondifferentiable minimization. Math. Program..

[22] Lasserre, J.B.: An explicit exact SDP relaxation for nonlinear 0-1 programs. In: Integer programming and combinatorial optimization (Utrecht, 2001), Lecture Notes in Compututer Science, vol. 2081, pp. 293–303. Springer, Berlin (2001)

[23] Laurent M, Poljak S, Balas E, Cornuejols G, Kannan R (1992). The metric polytope. Integer Programming and Combinatorial Optimization.

[24] Lovász L (1979). On the shannon capacity of a graph. IEEE Trans. Inf. Theory.

[25] Lovász L, Schrijver A (1991). Cones of matrices and set-functions and $$0$$-$$1$$ optimization. SIAM J. Optim..

[26] MOSEK ApS: The MOSEK optimization toolbox for MATLAB manual. Version 8.0. (2017). http://docs.mosek.com/8.0/toolbox/index.html

[27] Nguyen, T.H., Bui, T.: Graph coloring benchmark instances. https://turing.cs.hbg.psu.edu/txn131/graphcoloring.html. Last accessed 15 May 2019

[28] Rendl F, Rinaldi G, Wiegele A (2010). Solving max-cut to optimality by intersecting semidefinite and polyhedral relaxations. Math. Program..

[29] Rendl F, Sotirov R (2007). Bounds for the quadratic assignment problem using the bundle method. Math. Program..

[30] Robinson SM (1989). Bundle-based decomposition: conditions for convergence. Ann. l’I.H.P. Anal. non linéaire.

[31] Rockafellar RT (1970). Convex Analysis. Princeton Mathematical Series, No. 28.

[32] Sherali HD, Adams WP (1990). A hierarchy of relaxations between the continuous and convex hull representations for zero-one programming problems. SIAM J. Discrete Math..

[33] Toh KC, Todd MJ, Tütüncü RH (1999). SDPT3—a MATLAB software package for semidefinite programming, version 1.3. Optim. Methods Softw..

[34] Tütüncü RH, Toh KC, Todd MJ (2003). Solving semidefinite-quadratic-linear programs using SDPT3. Math. Program..

[35] Yang L, Sun D, Toh KC (2015). $${\rm SDPNAL}+$$: a majorized semismooth Newton-CG augmented Lagrangian method for semidefinite programming with nonnegative constraints. Math. Program. Comput..

